# Outcomes of the first meeting of the CAMEROON HIV RESEARCH FORUM (CAM-HERO)

**DOI:** 10.11604/pamj.2021.40.166.31058

**Published:** 2021-11-17

**Authors:** Boris Tchounga, Rogers Ajeh, Tatiana Djikeussi, Peter Vanes Ebasone, Patrice Tchendjou, Jean Anoubissi, Ezekiel Ngoufack Jagni Semengue, Charles Nkouanfack, Fabrice Youbi Kamgang, Marie Varloteaux, Boris Youngui Tchakounte, Felicite Naah Tabala, Benjamin Atanga, Leonie Simo, Jérôme Ateudjieu, Armel Zemsi, Lainsi Judith Nasah, Njie George Ngeke, Nicoline Ndiforkwah, Mireille Teno Bouseko, Falone Tientchou Sandjong, Emile Nforbih Shu, Madeleine Bakari, Yves Tiojio, Gilles Ndayisaba, Nyenti Annereke, Apungwa Cornelius Ntabe, Therese Abong Bwemba, Serge Clotaire Billong, Anne Cecile Zoung-Kanyi Bisseck, John Ditekemena, Anastase Dzudie

**Affiliations:** 1 Elizabeth Glaser Pediatric AIDS Foundation, Yaoundé, Cameroon,; 2 Clinical Research Education, Networking and Consultancy, Yaoundé, Cameroon,; 3 Faculty of Health Sciences, University of Buea, Buea, Cameroon,; 4 Department of Medicine, University of Cape Town, Cape Town, South Africa,; 5 National AIDS Control Committee, Ministry of Public Health, Yaoundé, Cameroon,; 6 International Reference Centre Chantal Biya (IRCCB), Yaoundé, Cameroon,; 7 HIV Day Hospital, Yaoundé Central Hospital, Yaoundé, Cameroon,; 8 Faculty of Medicine and Pharmaceutical Sciences, University of Dschang, Dschang, Cameroon,; 9 Division of the Fight against Diseases, Ministry of Public Health, Yaoundé, Cameroon,; 10 Cameroon Office, National Agency for Research on AIDS (ANRS), Yaoundé, Cameroon,; 11 Division of Health Operational Research, Ministry of Public Health, Yaoundé, Cameroon,; 12 Limbe Regional Hospital, Limbe, Cameroon,; 13 Bamenda Regional Hospital, Bamenda, Cameroon,; 14 Jamot Hospital, Yaoundé, Cameroon,; 15 Cameroon Bioethics Initiative, Yaoundé, Cameroon,; 16 National Ethics Committee, Yaoundé, Cameroon,; 17 Faculty of Medicine and Biomedical Sciences, University of Yaoundé I, Yaoundé, Cameroon,; 18 Service of Internal Medicine and Subspecialities, Douala General Hospital, Douala, Cameroon,; 19 Lown Scholars Program, Department of Global Health and Population, Harvard T. H. Chan School of Public Health, Boston, USA

**Keywords:** HIV, research, Cameroon, meeting report

## Abstract

Research is a vital component for the development of any country. In Cameroon, HIV Operational research is rapidly growing, however, it faces some intractable problems which can only be solved through an urgent, strategic, efficient, and collaborative approach involving key stakeholders. The Kribi meeting (09 and 10^th^ December 2020) brought together under the auspices of the Ministry of Public Health leading HIV research organisations and connected HIV researchers and actors from different sectors. These actors disseminated and discussed recent research findings and worked out mechanisms to advance HIV research development, developed new ideas and identified priority research areas, with emphasis on translational research. The official launching and consolidation of Cam-HERO was a critical step and it is hoped that these synergistic efforts will catalyse attainment of the 95-95-95 goals in Cameroon.

## Conference proceedings

### Introduction

**Figure 1:**
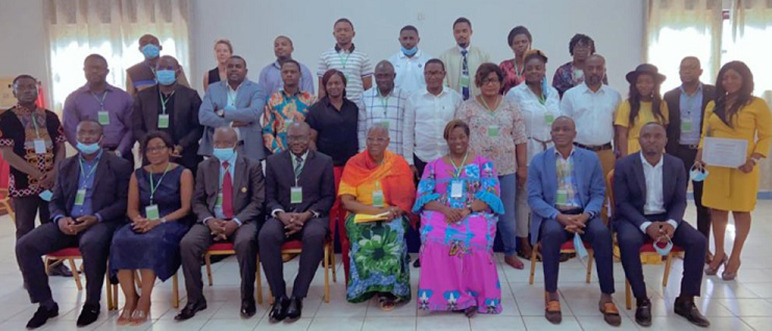
group photo during the 2020 edition of the Cameroon HIV Research Forum

Research is a critical catalyst of technological progress and countries’ development over the last century [1]. Health research is more efficient when its implementation is well planned, coordinated, and synergistic [2]. Poor planning, collaboration, and coordination in research may lead to several inconveniences including inefficient use of resources, duplication of efforts, and misplaced/mismatched research priorities that do not align with national and international priorities [3]. There is evidence of rapidly increasing HIV research in Cameroon. Although most of these researchers, work in collaboration with the Cameroon Ministry of Public Health, and the Ethics Committee, little has been done so far to encourage them to: 1) Work collaboratively and synergistically as research teams and with other actors on the field; 2) Meet up with their various challenges including those related to the development of new ideas and priority research areas, fostering of capacity building, and innovations; 3) Disseminate their research findings at the national level, including translation of research evidence into practice within the context. In this light, the main HIV research partners met in Kribi on the 9^th^ and 10^th^ of December 2020 to officially launch the Cameroon HIV/AIDS Operational Research Forum (Cam-HERO) under the patronage of the Ministry of Public Health (Figure 1). This included the Elizabeth Glaser Pediatric AIDS Foundation (EGPAF), the International epidemiology Databases to Evaluate AIDS/Clinical Research Education Networking and Consultancy research group (CRENC-IeDEA), the Agence Nationale de la Recherche Scientifique, Cameroon (Site ANRS), the Yaoundé Central Hospital HIV research team, the Chantal Biya International HIV research center, the National AIDS Control Committee (NACC), the National Ethics Committee and individual renowned HIV researchers.

### Day 1: 9^th^ December 2020

The meeting was officially opened by Prof. Anne Cecile Bissek, the Director of the Division of Operational Research (DROS) at the Cameroon Ministry of Public Health. She commended the Cam-HERO initiative and remarked that it was about time HIV research actors in the country come together to align their efforts in a coordinated manner towards helping the country to fight the HIV/AIDs epidemic in Cameroon. Prof Dzudie made an opening presentation of the Cam-HERO; its goals, core strategies, partners, and future perspectives. He emphasized the importance of connecting all HIV research actors, with the Ministry of Health and other relevant administrative bodies, and critically with ethics committees; to work out mechanisms of advancing HIV research development and innovation in Cameroon.

### Presentation of oral abstracts

Dr. Rogers Ajeh presented a description of the Cameroon IeDEA project. IeDEA had enrolled a cohort of over 13,000 people living with HIV/AIDS (PLHV) in Cameroon into its database three HIV clinics (Jamot Hospital, Limbe Regional Hospital, and Bamenda Regional Hospital).

Prof Dzudie then presented a cross-sectional analysis of hypertension in PLHV from the Cameroon IeDEA. In this study 1 of every [4]. PLHV aged 20 years or above had uncontrolled hypertension, a serious threat to the attainment of the HIV millennium goals. This highlighted the urgent need for task shifting of hypertension management to HIV care nurses.

Dr. Patrice Tchendjou presented an analysis from EGPAF entitled “HIV mother-to-child transmission in Cameroon: Early infant diagnosis positivity rates by entry point and key risk factors”. HIV positivity rate was higher (17.6%) at non-PMTCT entry points compared to 5.7% at PTMCT entry points. This highlighted the need to consider non-PMTCT entry points to increase the identification of HIV-positive children.

Dr. Armel Zemsi presented an analysis of the “HIV infection status of children under five years of age identified as tuberculosis (TB) presumptive in Cameroon”. The study revealed that 62% of children with a presumptive diagnosis of TB had an unknown HIV status. Presentation of CAM-HERO partners´ research portfolios

This section of the meeting was punctuated by the presentation of 03 research portfolios.

Prof Laura Guay, the vice-president of Global Research at EGPAF presented the mission and goals of EGPAF's research department. EGPAF aims to develop an extensive research portfolio that covers evaluations of different programme activities tailored to each country´s priorities with an overall goal to improve the outcomes of monitoring and treatment of HIV. Some studies in EGPAF´s portfolio include the INPUT and CONTACT intervention trials, TIPPI, POC EID, New Horizon, and Reach 95 evaluation studies.

Prof Kathryn Anastos, Principal Investigator of the IeDEA Central Africa [4] presented the IeDEA project, an international research consortium established in 2006 that collaborates to collect and define key variables, harmonize data, and implement methodology to effectively pool data as a cost-effective means of generating large data sets to address the high priority research questions and streamline HIV/AIDS research. The Central Africa-IeDEA (CA-IeDEA) cohort includes more than 86,000 patients who enrolled in HIV care at 21 participating sites, including 3 in Cameroon. Prof Anastos highlighted that their key challenges have been ensuring high-quality data collection and unstable human capital investment.

Dr Marie Varloteaux technical assistant at the ANRS Cameroon Site pointed out that the ARNS is an international medical and scientific research platform with a collaborative agenda to develop joint actions and share experiences. Their areas of collaboration include; medical research, national HIV policy development and capacity building among others.

Dr Charles Kouanfack, HIV PI of the Yaounde Central Hospital, presented an ANRS study entitled “Dolutegravir (DTG) versus Efavirenz (EFV) 400mg as a protocol for the initial treatment of HIV-infected patients in Cameroon (NAMSAL Trial)”. The study showed the confirmation of non-inferiority of DTG versus EFV-600 at week 48 as well as the non-inferiority of DTG versus EFV-400 at week 96. Dr Kouanfack equally suggested that DTG scale-up should be done with caution due to risk of weight gain.

At the end of day 1, discussions from experts highlighted three key points a) the urgent need to develop HIV research priorities in Cameroon, b) the importance of connecting all HIV research players under the patronage of the Ministry of Health, with support (including funding) from all other research institutions, both national and international c) the need of a very close collaboration between researchers and national, regional and institutional ethics committees.

### Day 2: 10^th^ December 2020

The second day started with a session dedicated to the definition of HIV research priorities for the attainment of the 95-95-95 targets in Cameroon.

### Identification and of Cameroon HIV research priorities

The methodology adopted for identifying and choosing the research priorities was the Delphi method steps approach 1) proposition of a list of research priorities by all actors, discussion and merging by the audience; 2) identification of the most critical priorities and 3) consensus to select research priorities. The NACC opened the presentation of research priorities. Dr Billong Serge, deputy permanent secretary of the NACC made a succinct presentation of the results of the fight against HIV in Cameroon for the first half of 2020 and research areas of interest for the NACC which are focused on the attainment of the three 95s. Dr Boris Tchounga presented priority research areas from an EGPAF initiated project named “Projet Atteindre 95”, an ongoing project that aims to reduce gaps and accelerate progress toward controlling the HIV epidemic by strengthening HIV service delivery, human resources for health, and the information documentation system at all levels of the health system. Dr Rogers Ajeh presented CRENC-IeDEA´s proposed research priority questions for each of the 95-95-95 targets and pertinent research questions for implementation science. The floor was opened for discussion and evaluation of research priorities proposed, identification of those that are important for advancing HIV/AIDS care in Cameroon and then picking by consensus 12 research priorities which will be streamlined by the researchers.

### Opportunities for effective collaboration between researchers, ethics committees and administrators

This session was opened by a presentation from the Vice President of the Cameroon National Ethics Committee, Dr Thérèse Abong Bwemba. The presentation covered a recap on fundamental ethical principles, the Cameroon National Ethics Committee´s mission, how to prepare and submit a research protocol and the impact on COVID-19 on their activities.

Mr Apungwa Cornelius from Cameroon Bioethics Initiative (CAMBIN) focused on ethics review in Cameroon before and after COVID-19. CAMBIN noted that Cameroonian researchers were deficit in research ethics training based on observations made from the quality of protocols submitted for review to them.

Mrs Naah Félicité from the DROS presented the Division´s mission, state of ethical and administrative authorization of HIV research projects in Cameroon, the institutional framework for implementing human health research in Cameroon and how to apply for administrative authorization from DROS. There was a significant gap between the number of ethical clearances (724) issued and the number of administrative authorisations (126). The DROS has streamlined the process for obtaining administrative clearance to a maximum of 2 weeks.

Discussions from the three presentations emphasized on the need of collaborations to avoid miss understandings.

The meeting ended with the award of certificates of appreciation to participants and a family picture to crown the day (Figure 1).

### Conclusion

Operational HIV research is rapidly progressing in Cameroon but facing some intractable problems which are inherent to research in Africa. These problems can be addressed through a strategic, efficient and collaborative approach involving all key stakeholders. The Kribi meeting brought together health renowned research organizations and connected HIV research players from different sectors. These actors committed to work out mechanisms to advance HIV research development, develop new ideas and priority research areas, and disseminate their research findings, with emphasis on the translation of research evidence into practices to improve HIV care outcomes in Cameroon.

## References

[1] World Health Organization (2012). Research and development to meet health needs in developing countries: strengthening global financing and coordination: report of the consultative expert working group on research and development: financing and coordination. World Health Organization.

[2] Spaak M, Cipriano M, Alla F, Benamouzig D (2021). Health services research in France: bridging the gap between academia and policymaking. Eur J Public Health.

[3] Gulati R, Wohlgezogen F, Zhelyazkov P (2012). The two facets of collaboration: Cooperation and coordination in strategic alliances. Acad Manag Ann.

[4] Central Africa IeDEA Improving health outcomes of individuals living with HIV/AIDS.

